# Discovery of the Key Mutation Site Influencing the Thermostability of *Thermomyces lanuginosus* Lipase by Rosetta Design Programs

**DOI:** 10.3390/ijms23168963

**Published:** 2022-08-11

**Authors:** Enheng Zhu, Xia Xiang, Sidi Wan, Huabiao Miao, Nanyu Han, Zunxi Huang

**Affiliations:** 1 School of Life Sciences, Yunnan Normal University, Kunming 650500, China; 2 Engineering Research Center of Sustainable and Utilization of Biomass Energy, Ministry of Education, Yunnan Normal University, Kunming 650500, China; 3 Key Laboratory of Yunnan for Biomass Energy and Biotechnology of Environment, Yunnan Normal University, Kunming 650500, China; 4 Key Laboratory of Enzyme Engineering, Yunnan Normal University, Kunming 650500, China

**Keywords:** lipase, thermostability, Rosetta, Gibbs free energy

## Abstract

Lipases are remarkable biocatalysts and are broadly applied in many industry fields because of their versatile catalytic capabilities. Considering the harsh biotechnological treatment of industrial processes, the activities of lipase products are required to be maintained under extreme conditions. In our current study, Gibbs free energy calculations were performed to predict potent thermostable *Thermomyces lanuginosus* lipase (TLL) variants by Rosetta design programs. The calculating results suggest that engineering on R209 may greatly influence TLL thermostability. Accordingly, ten TLL mutants substituted R209 were generated and verified. We demonstrate that three out of ten mutants (R209H, R209M, and R209I) exhibit increased optimum reaction temperatures, melting temperatures, and thermal tolerances. Based on molecular dynamics simulation analysis, we show that the stable hydrogen bonding interaction between H198 and N247 stabilizes the local configuration of the 250-loop in the three R209 mutants, which may further contribute to higher rigidity and improved enzymatic thermostability. Our study provides novel insights into a single residue, R209, and the 250-loop, which were reported for the first time in modulating the thermostability of TLL. Additionally, the resultant R209 variants generated in this study might be promising candidates for future-industrial applications.

## 1. Introduction

Lipases (EC 3.1.1.3) are remarkable biocatalysts that can not only catalyze the hydrolysis of natural oil into mono-, di-glycerides, fatty acid, and glycerol, but also facilitate esterification, transesterification, alcoholysis, acidolysis, and many other reactions [1]. In addition to the versatile catalytic capabilities, lipases are also important for their enantioselectivity, regional selectivity, high catalytic activity, and rare catalytic side reactions [2,3]. Therefore, lipases are widely used in textiles, detergents, food production, pharmaceutical synthesis, and the biofuel industry [4,5]. Taking the harsh conditions in the industrial processes into consideration, the activity and stability of lipase products are required to be stable in the process of severe biotechnological treatment. To this aim, lipase products with superior thermostability, alkaline/acid tolerance, and high catalytic efficiency for industrial application are in high demand.

Lipases can be extracted from a broad range of sources, such as plants, animals, and microorganisms. Among them, lipases from microorganisms are more advantageous due to their ease of genetic manipulation, rapid growth, high yields, variable catalytic activities, and superior stability comparing lipases from plants and animals [6]. Accordingly, microbial lipases play a crucial role in industrial processes. Table 1 listed the reported microbial lipases with their thermal properties. Among them, lipase from *Thermomyces lanuginosus* (TLL) has displayed fast reaction speed, excellent catalytic activity and thermal stability, for which reasons it has become a popular commercial biocatalyst [7]. However, the optimal reaction temperature of TLL is lower than 50 °C [8], which cannot meet the requirement of most industrial standards. Therefore, our research aim is to improve the thermostability of TLL for being a qualified commercial product in industry.

Protein engineering is a powerful tool that can modify various desirable properties of proteins. To achieve this aim, three strategies have been proposed: (1) directed evolution, (2) structural-based rational design, and (3) computer-assisted design (CAD) [19]. Traditional directed evolution mimics the Darwinian evolution, which requires the construction of a mutational library with abundant random mutations followed by a large amount of selecting and verifying work to enrich the final desired enzymes. However, directed evolution is more time and economic consuming. To this end, structural-based rational design depending on the deep understanding of the protein structure–function relationship and CAD achieved by calculating the protein movements, etc., has been widely applied [20,21,22,23]. For example, structural analysis and molecular dynamics simulation both indicated that introducing a proline (V213P) to lipase from *Yarrowia lipolytica* may enhance enzymatic stability. Experimental results revealed V213P with improved thermostability: the optimum temperature of V213P was increased by 5 °C, and its catalytic activity was comparable to the wild-type [10]. Han et al. modified the surface charged residues of TLL predicted by CAD, and the multiple resultant TLL variants with both improved catalytic activity and biodiesel production proved the effectiveness of the CAD method [24]. Reetz et al. constructed *Bacillus subtilis* lipase variants using the B-FIT method and finally obtained lipases with a 500-fold improvement in enzyme half-life at 55 °C [25]. Compared to the directed evolution, structural-based rational design and CAD offer a more efficient and specific strategy in protein engineering.

To improve the thermostability of an enzyme, structural-based rational design and CAD are both effective. Based on structural analysis and multiple sequence alignment (MSA), flexible regions will be substituted with the conserved amino acids and stabilized recombinants will be generated: this is the most common approach for rational design to build a stable recombinant [26]. For the CAD approach, the energy changes of wild-type and mutants can be efficiently calculated based on the fundamental theories of physics and chemistry, and the calculating results will direct researchers to construct reliable enzyme variants [27].

Rosetta ddg_monomer and Rosetta Cartesian_ddG are two commonly used Gibbs free energy calculating programs provided by Rosetta design [28,29], and they have been widely and successfully applied in improving protein stability [29,30]. In this study, the Gibbs free energy changes of TLL and its single saturated mutants were calculated using Rosetta ddg_monomer and Cartesian_ddG. Our previous study on TLL discovered that substitutions on the surface charged residues can elevate TLL-specific activity and biodiesel yield [24]; thus, substitutions on charged residues predicted with enhanced stability by both programs were sorted and compared. We discovered that substituting residue R209 may fundamentally influence the TLL stability after comparing the Gibbs free energy changing results. More importantly, three R209 variants were verified with improved thermostability experimentally, and this computational design strategy may pave a new way for future study.

## 2. Results

### 2.1. Substituting R209 May Improve TLL Stability Predicted by Both Rosetta Programs

Our previous study discovered that substitutions on the surface charged residues of TLL can elevate its specific activity and biodiesel yield [24]. In this study, we focus on the predicted stabilized substitutions of the charged amino acids. The Gibbs free energy changing results from Rosetta ddg_monomer and Cartesian_ddG calculations were compared and substitutions on seven charged residues were predicted to be stabilized variants by both computational programs (ΔΔG < 0), including substitutions on D111, D158, D165, R209, R232, E239, and D254. Interestingly, substitutions on R209 are of remarkable potential that may improve TLL stability, as supported by fourteen R209 mutants with negative ΔΔG values predicted from Rosetta ddg_monomer and thirteen R209 mutants with negative ΔΔG values predicted from Rosetta Cartesian_ddG (Table S1). More importantly, ten overlapping stabilized R209 mutants were reported from both programs. For the remaining charged residues, the numbers of overlapped stabilized substitutions predicted by both programs are less than eight (Table S1). Based on the Gibbs free energy changing results, we plan to construct the following ten R209 mutants for the subsequent experimental validation, including R209A, R209E, R209H, R209K, R209M, R209P, R209T, R209I, R209Q, and R209V.

### 2.2. Construction and Characterization of the TLL Mutants

**Figure 1:**
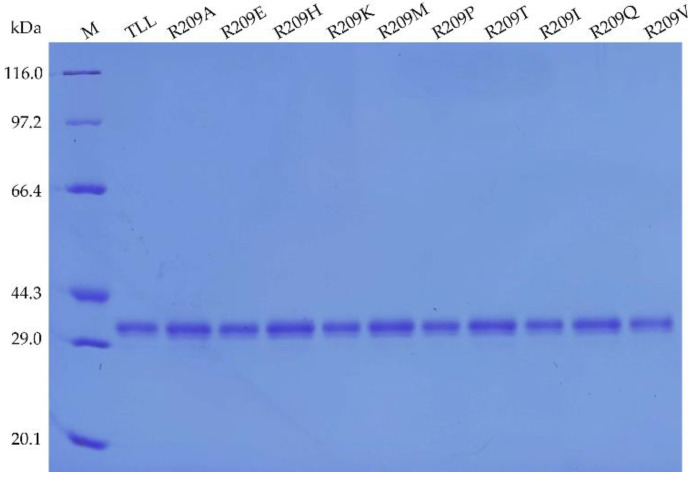
SDS-PAGE analysis of TLL and the ten R209 mutants.

As shown in the SDS-PAGE result (Figure 1), the ten purified R209 TLL mutants displayed similar molecular weight as TLL (30.05 kDa). The specific activities of purified TLL, R209A, R209E, R209H, R209K, R209M, R209P, R209T, R209I, R209Q, and R209V were 298.28, 406.91, 270.48, 310.50, 380.17, 302.49, 368.67, 333.67, 283.13, 292.51, and 315.18 U/mg, respectively.

### 2.3. Thermostability of TLL and the Ten R209 Mutants

**Figure 2:**
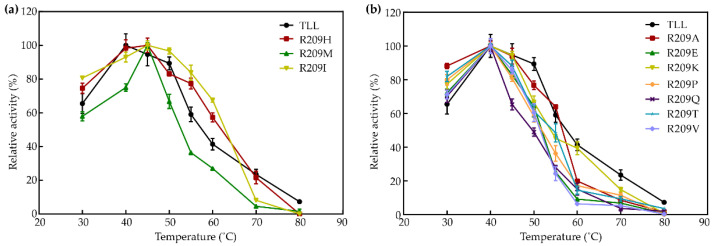
Optimal reaction temperature of TLL and ten R209 mutants. (**a**) R209 mutants with increased optimal reaction temperature; (**b**) R209 mutants revealed with the same optimal reaction temperature.

To compare the thermostability between TLL and the ten R209 mutants, optimal reaction temperatures (T_opt_), thermal tolerance, and melting temperature (T_m_) were assayed. T_opt_ of TLL was 40 °C, while T_opt_ of three R209 mutants (R209H, R209M, and R209I) was increased by 5 °C (Figure 2a). T_opt_ values of the other seven R209 mutants were the same as T_opt_ of TLL (Figure 2b).

**Figure 3:**
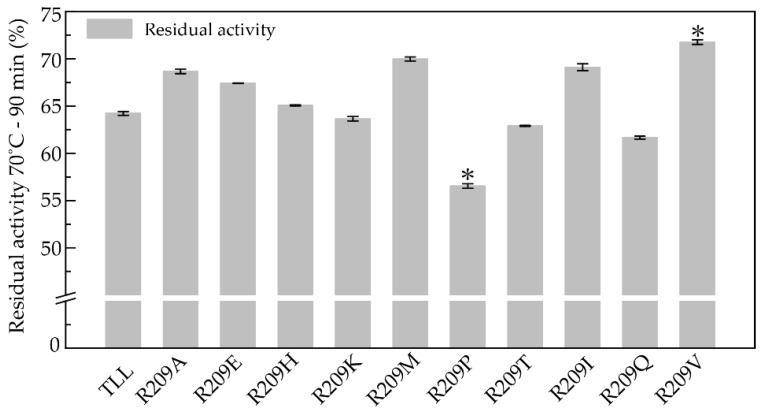
Residual activities of TLL and the ten R209 mutants. The residual activity was tested by incubating all lipases at 70 °C for 90 min. R209 mutants with one star indicate the differences of residual activity are significant at the 0.05 level.

Thermal tolerance was assessed by measuring the residual activity of TLL and R209-modulating candidates after incubation at 70 °C for 90 min. The thermal tolerances of the three R209 mutants which displayed increased T_opt_ values were stronger than that of TLL. After incubation at 70 °C for 90 min, enhanced residual activities of R209H, R209M, and R209I were observed compared to TLL (Figure 3). In addition, enhanced residual activities of another three mutants (R209V, R209A, and R209E) were also detected (Figure 3). The residual activities of R209V, R209A, and R209E were 72%, 69%, and 67.4% after incubation at 70 °C for 90 min, respectively, while TLL remained at 64% residual activity at the same condition. Despite the rest four R209 mutants (R209K, R209P, R209Q, and R109T) retained more than half of their initial activities after incubation at 70 °C for 90 min, their thermal tolerances were still weaker than that of TLL (Figure 3).

**Figure 4:**
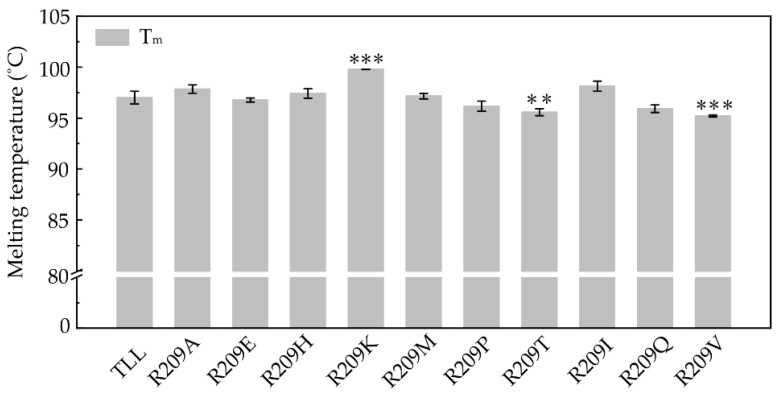
Melting temperatures (T_m_) of TLL and ten R209 mutants. R209 mutants with two and three stars indicate the differences in T_m_ values are significant at the 0.01 and 0.001 levels, respectively.

The melting temperatures (T_m_) were determined using differential scanning calorimetry (DSC). The apparent T_m_ value of TLL was 97 °C (Figure 4). Using this as the basal line, the T_m_ values of five R209 mutants (R209A, R209H, R209K, R209M, and R209I) were increased by 1, 0.4, 3, 0.2, and 1.1 °C compared to TLL, respectively (Figure 4). R209K exhibited the highest melting temperature (100 °C). More interestingly, it is noticed that R209H, R209M, and R209I with increased T_opt_ values and improved thermal tolerances also displayed elevated T_m_ values.

### 2.4. Kinetic Analysis of TLL and the Ten R209 Mutants

The kinetic parameters were determined at 37.0 °C and pH 9.0 with pNPP as the substrate (Table 2). Kinetic measurements showed that the apparent *K*_m_ values of R209E, R209P, and R209V decreased as compared with that of TLL. The smaller Michaelis constant (*K*_m_) indicates increased affinity of the substrate (pNPP), while R209A, R209H, R209K, R209M, R209T, R209I, and R209Q showed decreased affinity compared with TLL. Kinetic parameter *k*_cat_/*K*_m_ indicates the catalytic efficiency of an enzyme. R209E, R209K, R209P, and R209T displayed improved catalytic efficiency compared with that of TLL, due to their larger *k*_cat_/*K*_m_ values. For the three R209 mutants (R209H, R209M, and R209I) with improved thermostability, they all exhibited reduced substrate binding affinities and catalytic efficiencies, and which may on account of their more rigid configurations.

### 2.5. Improved Thermostability Explored by Molecular Dynamics Simulations

**Figure 5:**
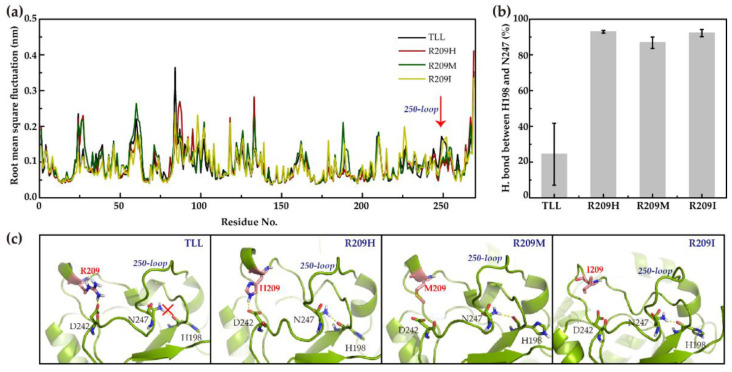
RMSF and hydrogen bonding interpretation of TLL and three R209 mutants. (**a**) Illustrates the root mean square fluctuation (RMSF) of TLL and the three R209 mutants during the simulations; (**b**) displays the hydrogen bond forming probability between H198 and N247 in the TLL and three R209 mutants during the entire simulations; (**c**) shows the structures of TLL, R209H, R209M, and R209I, respectively. Hydrogen bonding interaction and salt bridge interaction were labeled in yellow dash lines, residue 209 was labeled in red, and its carbon atoms are colored in salmon.

To explore the mechanism of the improved thermostability of the three R209 mutants, molecular dynamics (MD) simulations of TLL, R209H, R209M, and R209I were performed. The root mean square fluctuation (RMSF) reflects the flexibility of each residue during simulations. Comparing RMSF values of TLL and the three R209 mutants, residues of the 250-loop (residues 244–252) in TLL were discovered with higher RMSF values than those in the three R209 mutants (Figure 5a), indicating enhanced flexibility of the 250-loop in the TLL. To explore the reason for the flexibility changes, hydrogen bonding interactions of residues located on the 250-loop of TLL and the three R209 mutants were monitored. It is discovered that the probability of forming a hydrogen bond between H198 and N247 in R209H, R209M, and R209I were 93%, 87%, and 92%, respectively. However, this value decreased to 24% in TLL, indicating a loose connection between H198 and N247 of TLL (Figure 5b). The substituted residue R209 is located at a loop configuration in the TLL crystal structure, and its guanidine side-chain group formed a strong salt bridge interaction with the acyl group of D242 (Figure 5c). It is speculated that this salt bridge interaction may influence the H198-N247 hydrogen bond formation, resulting in a more flexible configuration of the 250-loop in TLL. On the contrary, the stable hydrogen bond interaction between H198 and N247 in the three R209 mutants enhances the stability of the 250-loop, which may give rise to the improved thermostability of the three R209 mutants (Figure 5c).

**Figure 6:**
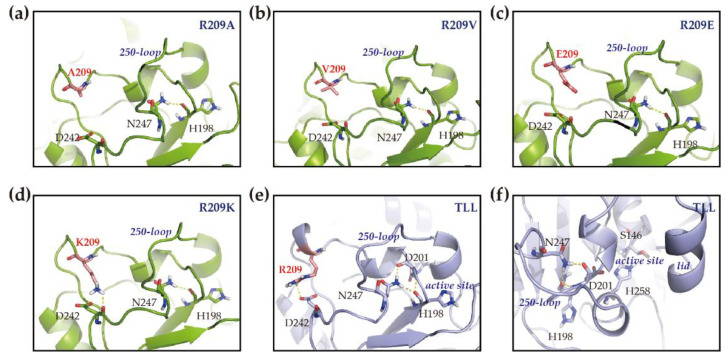
Structural modeling of R209 mutants and active site illustration of TLL. (**a**–**d**) Show the structures of R209A, R209V, R209E, and R209K, respectively; (**e**) displays the hydrogen bonding between D201 in the catalytic triad and nearby residues; (**f**) shows the relative position of the 250-loop, active site, and the lid domain of the TLL. Hydrogen bonding interaction and salt bridge interaction were labeled in yellow dash lines, residue 209 was labeled in red and its carbon atoms are colored in salmon.

Similarly, based on the modeling structure of R209A, R209V, R209E, and R209K, the hydrogen bonding interactions between H198 and N247 of the 250-loop were also discovered (Figure 6a–d). The active site of TLL contains a typical catalytic triad (S146•••D201•••H258). D201 in the active site was discovered that can interact with H198 and N247 with polar contact (Figure 6e). Moreover, the catalytic triad is located underneath the 250-loop, and the 250-loop is located on the opposite side of the lid domain (Figure 6f). Since the lid domain will transfer from the closed conformation to the open conformation when the substrate binds to the active site of lipase, it is of great importance to maintain the configuration of the active site for further catalytic reaction, and the stability of the 250-loop may play a pivotal role that contributes to higher rigidity of lipase.

## 3. Discussion

Enzyme products for industrial application are generally required to possess superior thermostability and catalytic activity upon the harsh biotechnological treatment. With the development of computer technology in the past 30 years, designing enzymes with desirable properties based on computational calculation is constantly emerging. In this study, the accuracy and efficiency of protein engineering using the CAD method are evidenced by the three promising R209 variants which are generated through the CAD method. Previously, Wu et al. employed the FRESCO workflow which consolidated multiple computational predicting results to design xylanase mutants for industry use. With this method, they obtained the best xylanase variant of which the apparent melting temperature was increased by 14 °C [27]. Taken together, these results suggest the combination of computational programs/modules can effectively improve enzymatic property to a larger extent. In our study, we also combined and compared the Gibbs free energy predicting results calculated by two programs (Rosetta ddg_monomer and Rosetta Cartesian_ddG). The three resultant lipase R209H, R209M, and R209I with improved optimal reaction temperatures, melting temperatures, and thermal tolerances support that combining the Gibbs free energy calculating results predicted by Rosetta ddg_monomer and Rosetta Cartesian_ddG is an effective approach to construct stabilized enzyme variants.

In the current study, we focus on modulating charged residues based on our previous finding which shows that substitutions on the surface charged residues of TLL can elevate the specific activities and biodiesel yields [24]. Thermal tolerance and high activity are both required for enzymes that are used in industrial applications. Therefore, our study on TLL charged residue modification might not only improve its activity, but also enhance its thermal tolerance, which will meet the high industrial standards. It is not surprising that the specific activities of most R209 mutants are higher than that of TLL, smoothing our path to select TLL variants with improved thermostability. Generally, substitutions with negative ΔΔG values are classified as stable mutants. In Rosetta ddg_monomer calculation, 898 substitutions were calculated with negative ΔΔG values, while 692 substitutions were calculated with negative ΔΔG values based on Rosetta Catesian_ddG prediction. Although the structural-based rational design and CAD protein engineering methods are more efficient than the directed evolution, constructing more than 600 enzyme variants in the experimental validation is still time and economic consuming. Our earlier findings guided us to narrow down the experimental validating work and shortlist the most desirable variants.

Although three R209 mutants with improved thermostability were proposed in this study, their catalytic efficiencies were not as good as the wild-type TLL. D201 in the catalytic triad forms polar contact with N247 of the 250-loop. Although the rigidity of the 250-loop in the three R209 mutants enhances thermostability, it also leads to mediocre performance in catalytic efficiency. Many studies have shown that enzymes with high thermostability are more rigid in structure and display weaker activity or catalytic efficiency. Thus, it is of great challenge to obtain enzymes with improved thermostability, specific activity, and catalytic efficiency simultaneously [31]. In our study, both R209E and R209K were identified with significantly increased *k*_cat_/*K*_m_ values, indicating that the comparable or improved catalytic efficiency of TLL may be controlled by its electrostatic properties.

Although the R209 mutants with improved thermostability displayed decreased catalytic efficiency, the excellent express yields of TLL are still able to compensate for the decrement in catalytic efficiency. TLL was characterized by higher yields than many other heat-resistant lipases: the enzyme activity of TLL after fermentation in a 150 mL test tube is closed to 1000 U/mL, while this value is only 400 U/mL in another thermal stable lipase WT-L2 from *Bacillus* sp. L2 [16,32]. In addition, when TLL was fermented in a 50 L fermenter in industry, its enzyme activity in the supernatant could reach 30,000 U/mL, which meets most of the conditions for the industrial production [33]. Therefore, TLL is a promising commercial biocatalyst for further modulating.

## 4. Materials and Methods

### 4.1. Materials

Site-directed mutagenesis kit was purchased from Trans Gen (Beijing, China). Restriction endonuclease (EcoR I, Not I), DNA marker, and protein marker were purchased from TaKaRa (Otsu, Japan). *Pichia pastoris* GS115 cells were purchased from Invitrogen (Shanghai, China). Mutant primers were synthesized by Shuoqing (Kunming, China). The *Thermomyces lanuginosus* lipase gene (*TLL*) cloned in the pPIC9K vector was deposited in our laboratory. All other chemicals were commercially available and of analytical grade.

### 4.2. Gene Cloning and Site-Directed Mutagenesis

TLL gene linked in pPIC9K was used as the template, and the point mutations in all of the TLL mutants were introduced using the Fast MultiSite Mutagenesis System according to the manufacturer’s instructors. Forward and reverse primers for TLL and the genes of the TLL mutants are listed in Table S2. The PCR procedure was set as: 5 min at 94 °C, followed by 30 cycles of 30 s at 94 °C, 2 min at 55 °C, and 10 min at 72 °C.

### 4.3. Protein Expression and Purification

Plasmids of wild-type lipase and mutants were linearized by *Sal I* and transformed into *Pichia pastoris* GS115 by electroporation individually. Recombinants were screened with 200 µg/mL G-418 YPD (yeast extract peptone dextrose) medium, which was then grown for 2 days at 30 °C in BMGY liquid media. The thallus was transferred to BMMY liquid medium at 30 °C for 2 days for the induction of protein expression. All enzymes labeled with His-tag at the N-terminus were purified by Ni-NTA agarose column. Bradford protein assay kit was used to measure enzyme concentrations.

### 4.4. Assessment of Lipase Activity

The lipase activity was measured using 4-nitrophenyl palmitate (pNPP) as the substrate. One unit (IU) of lipase activity was defined as the amount of enzyme that releases 1 μmol of p-nitrophenol per minute [34]. Thermostability was assayed by measuring the residual enzyme activity after incubation at 70 °C for 90 min under the optimal pH (9.0). Kinetic activity was assayed at 37 °C and pH 9.0 in Tris-HCl buffer (100 mM) with pNPP concentration of 0.08~10.00 mM. Kinetic parameters *K*_m_, *V*_max_, and *k*_cat_ were calculated by fitting to the Michaelis–Menten function [35].

### 4.5. Gibbs Free Energy Calculations Using Rosetta ddg_monomer and Cartesian_ddG

The crystal structure of TLL (1DT3) was used as the wild-type template for Gibbs free energy calculation [36]. The Rosetta ddg_monomer and Cartesian_ddG programs were used to calculate the Gibbs free energy changes (ΔΔG) between wild-type TLL and all of the possible single substitutions of the TLL (5111 in total) [15,16]. Default parameters were applied in both two calculations.

### 4.6. MD Simulation Details

The X-ray structure of TLL was taken from PDB 1DT3 and three TLL variants (R209H, R209M, and R209I) were built by SWISS-MODEL sever [37]. Normal MD simulations of TLL and the three R209 variants were performed at 45 °C. After 1000-step energy minimization, all systems were equilibrated for 5 ns followed by 10 ns simulation. Sodium and chloride ions were added with a concentration of 100 mM to neutralize the systems. The GROMACS program suite version 4.5.7 and Amber ff99SB force field were applied in all simulations [38]. All simulations were performed in an isothermal–isobaric ensemble (45 °C, 1 bar).

## 5. Conclusions

In this study, Gibbs free energy calculations were conducted to predict possible thermostable TLL variants, and the overlapping predicting results from two programs (Rosetta ddg_monomer and Rosetta Cartesian_ddG) were selected. Based on our earlier finding that indicates modulating TLL surface charged residues might improve its activity and biodiesel yield, we only focus on substitutions of the charged residues. One of the significant findings is that substituting on a key residue R209 may greatly influence TLL thermostability. Accordingly, ten TLL variants (R209A, R209E, R209H, R209K, R209 M, R209P, R209T, R209I, R209Q, and R209V) were generated and verified by a series of biological experiments. Interestingly, three out of ten mutants (R209H, R209M, and R209I) displayed increased optimum reaction temperatures, melting temperatures, and thermal tolerances. On the basis of MD simulation results, it revealed that the hydrogen bonding interaction between H198 and N247 stabilizes the local 250-loop configuration, and the stability of the 250-loop is of great importance in contributing to the improved thermostability of the three R209 mutants. It is the first time that identified substitutions on R209 can improve the thermostability of TLL. The resultant R209 mutants (R209H, R209M, and R209I) generated in this study will be promising candidates for industrial application, and the strategy of combining different computational programs will lead a broad way for future enzyme design.

## Figures and Tables

**Table 1:** Sources and thermal stability of microbial lipases.

Lipase	Source	T_opt_	T_1/2_	References
TLL	*Thermomyces lanuginosus*	40 °C	30 min (80 °C)	this article
PCL	*Penicillium cyclopium*	25 °C	4.70 min (45 °C)	[9]
LIP2	*Yarrowia lipolytica*	37 °C	~75 min (50 °C)	[10]
CRL	*Candida rugosa*	40 °C	4.2 min (50 °C)	[11]
RCL	*Rhizopus chinensis*	40 °C	4 min (60 °C)	[12]
Lipr27RCL	*Rhizopus chinensis*	40 °C	~25 min (60 °C)	[13]
SML	*Serratia marcescens*	42 °C	60 min (67 °C)	[14]
ELBn12	*Enterobacter* sp. Bn12	60 °C	~20 min (70 °C)	[15]
Wt-L2	*Bacillus* sp. L2	70 °C	8 h (60 °C)	[16]
RD	*Rhodothermus marinus*	70 °C	~1 h (75 °C)	[17]
CalB	*Candida antarctica*	N.A	49.3 min (50 °C)	[18]

**Table 2:** Kinetics of TLL and the ten R209 mutants.

Enzymes	*V*_max_ (μmol/min/mg)	*K*_m_ (mM)	*k*_cat_ (/s)	*k*_cat_/*K*_m_ (/s/mM)
TLL	297.36 ± 18.76	0.16 ± 0.03	850.09 ± 11.84	5313.06 ± 74.02
R209A	384.35 ± 19.28	0.23 ± 0.03 *	1220.16 ± 42.96	5305.04 ± 186.77
R209E	300.52 ± 15.84	0.14 ± 0.02	1230.63 ± 27.58	8790.21 ± 197.02 ***
R209H	330.01 ± 17.77	0.18 ± 0.02	790.25 ± 22.66	4390.28 ± 125.89 ***
R209K	345.37 ± 18.78	0.17 ± 0.02	1096.41 ± 14.02	6499.47 ± 82.49 ***
R209M	319.30 ± 18.57	0.19 ± 0.03	764.61 ± 17.56	4024.26 ± 92.42 ***
R209P	338.70 ± 16.52	0.16 ± 0.02	1386.98 ± 12.85	8668.63 ± 80.34 ***
R209T	343.89 ± 22.43	0.19 ± 0.03	1091.71 ± 27.64	5745.84 ± 145.51
R209I	302.90 ± 13.22	0.18 ± 0.02	767.22 ± 22.81	4262.33 ± 126.74 ***
R209Q	354.42 ± 20.54	0.23 ± 0.03 *	787.60 ± 7.98	3424.34 ± 34.69 ***
R209V	259.78 ± 15.75	0.14 ± 0.02	712.70 ± 8.11	5090.71 ± 57.88

*^/^*** R209 mutants with one and three stars indicate the differences of *K*_m_ or *k*_cat_/*K*_m_ values are significant at the 0.05 and 0.001 levels, respectively.

## Data Availability

Data is contained within the article or supplementary material.

## Supplementary Materials

The following supporting information can be downloaded at: https://www.mdpi.com/article/10.3390/ijms23168963/s1.
