# Supplementary material for: Discovery of the Key Mutation Site Influencing the Thermostability of Thermomyces lanuginosus Lipase by Rosetta Design Programs
Source: Int J Mol Sci. 2022 Aug 11;23(16):8963. doi: 10.3390/ijms23168963 (PMC9408933; doi:10.3390/ijms23168963)
Supplement: Supplementary file 1 [file ijms-23-08963-s001.zip › ijms-1841987-supplementary.pdf]

Table S1 Stable substitutions of charged residues predicted by Rosetta programs.

Charged Residues	Rosetta Cartesian_ddG (ΔΔG<0)		Rosstta ddg_monomer (ΔΔG<0)		Overlapped substitutions
D111	D111A	-1.056	D111A	-2.802	D111A
	D111K	-0.165	D111K	-4.101	D111K
	D111L	-0.582	D111L	-2.695	D111L
	D111R	-0.776	D111R	-1.955	D111R
	D111V	-0.209	D111V	-1.264	D111V
	D111W	-0.352	D111W	-1.653	D111W
	D111Y	-0.342	D111Y	-2.14	D111Y
			D111M	-2.355	
			D111N	-0.804	
			D111H	-1.323	
			D111F	-2.102	
			D111I	-2.309	
			D111S	-1.449	
			D111T	-0.333	
			D111E	-1.974	
D158	D158M	-1.688	D158M	-2.259	D158M
	D158Q	-0.245	D158Q	-1.438	D158Q
	D158V	-0.801	D158V	-1.659	D158V
	D158F	-1.383	D158F	-3.955	D158F
	D158L	-0.362	D158L	-2.296	D158L
	D158E	-0.254	D158N	-0.776	
			D158A	-1.327	
			D158H	-3.365	
			D158R	-0.557	
			D158S	-2.126	
			D158T	-0.297	
			D158W	-3.29	
			D158Y	-3.145	
D165	D165Q	-0.313	D165Q	-0.903	D165Q
	D165E	-1.306	D165A	-1.674	
			D165F	-0.613	
			D165H	-0.298	
			D165I	-0.785	

			D165M	-0.398	
			D165N	-0.291	
			D165P	-0.766	
			D165R	-0.73	
			D165T	-0.46	
			D165V	-0.741	
			D165W	-0.635	
R209	R209A	-0.501	R209A	-0.821	R209A
	R209E	-0.15	R209E	-0.503	R209E
	R209H	-0.205	R209H	-1.646	R209H
	R209I	-0.188	R209I	-1.194	R209I
	R209K	-0.01	R209K	-0.72	R209K
	R209M	-0.197	R209M	-0.901	R209M
	R209P	-3.149	R209P	-2.275	R209P
	R209Q	-1.959	R209Q	-0.307	R209Q
	R209T	-1.229	R209T	-1.496	R209T
	R209V	-0.694	R209V	-1.147	R209V
	R209C	-0.221	R209W	-0.244	
	R209L	-1.536	R209Y	-0.487	
	R209S	-0.931	R209D	-0.048	
			R209N	-1.135	
R232	R232A	-1.16	R232A	-0.757	R232A
	R232F	-0.508	R232F	-2.756	R232F
	R232H	-0.214	R232H	-2.175	R232H
	R232P	-2.249	R232P	-1.797	R232P
	R232Q	-0.303	R232Q	-1.452	R232Q
	R232V	-1.463	R232V	-0.886	R232V
	R232W	-0.253	R232W	-4.209	R232W
	R232Y	-0.847	R232Y	-2.171	R232Y
	R232K	-1.836	R232E	-0.347	
	R232I	-0.493	R232N	-0.34	
			R232S	-1.021	
			R232T	-0.586	
E239	E239L	-0.618	E239L	-1.069	E239L
	E239T	-0.721	E239T	-0.793	E239T
	E239V	-0.426	E239V	-0.045	E239V

	E239D	-1.351	E239F	-1.746	
			E239A	-0.099	
			E239I	-0.138	
			E239K	-0.07	
			E239M	-1.108	
			E239Q	-1.241	
			E239R	-0.009	
			E239W	-0.654	
			E239Y	-1.666	
D254	D254F	-0.426	D254F	-3.824	D254F
	D254Y	-0.357	D254Y	-4.248	D254Y
			D254C	-0.348	
			D254G	-1.421	
			D254H	-3.679	
			D254A	-2.563	
			D254K	-3.463	
			D254L	-2.434	
			D254M	-1.67	
			D254N	-1.55	
			D254Q	-0.747	
			D254T	-1.249	
			D254V	-3.707	
			D254W	-3.189	

Table S2. Oligonucleotide primers for *TLL* and genes of the ten R209 mutants.

Primers	Primer sequences
TLL-forward	5′-GAGGTCTCGCAGGATCTGTTTAACCAG-3′
TLL-reverse	5′- AAGACATGTTCCAATTAAACCGAAGTA-3′
R209A-forward	5′-CCTAGACTCCCTCCA**GC**CGAGTTCGGTTA-3′
R209A-reverse	5′-**GC**TGGAGGGAGTCTAGGGACAATATCATTGGT-3′
R209E-forward	5′-CCTAGACTCCCTCCA**GAA**GAGTTCGGTTAC-3′
R209E-reverse	5′-**TTC**TGGAGGGAGTCTAGGGACAATATCATT-3′
R209H-forward	5′-CCTAGACTCCCTCC**A**CACGAGTTCGGTTA-3′
R209H-reverse	5′-G**T**GTGGAGGGAGTCTAGGGACAATATCATT-3′
R209K-forward	5′-CCTAGACTCCCTCCA**AAG**GAGTTCGGTTAC-3′
R209K-reverse	5′-**CTT**TGGAGGGAGTCTAGGGACAATATCATT-3′
R209M-forward	5′-CCTAGACTCCCTCCA**ATG**GAGTTCGGTTACA-3′
R209M-reverse	5′-**CAT**TGGAGGGAGTCTAGGGACAATATCATT-3′
R209P-forward	5′-CCTAGACTCCCTCCAC**C**CGAGTTCGGT-3′
R209P-reverse	5′-**G**GTGGAGGGAGTCTAGGGACAATATCA-3′
R209T-forward	5′-CCTAGACTCCCTCCA**AC**CGAGTTCGGTTA-3′
R209T-reverse	5′-**GT**TGGAGGGAGTCTAGGGACAATATCATTGGT-3′
R209I-forward	5′-CCTAGACTCCCTCCA**AT**CGAGTTCGGTTA-3′
R209I-reverse	5′-**AT**TGGAGGGAGTCTAGGGACAATATCATTGGT-3′
R209Q-forward	5′-CCTAGACTCCCTCCAC**AA**GAGTTCGGTTACA-3′
R209Q-reverse	5′-**TT**GTGGAGGGAGTCTAGGGACAATATCATT-3′
R209V-forward	5′-CCTAGACTCCCTCCA**GT**CGAGTTCGGTTA-3′
R209V-reverse	5′-**AC**TGGAGGGAGTCTAGGGACAATATCATTGGT-3′

Nucleotide resulting in the desired mutation is underlined and in bold
